# 

**Published:** 1965-10

**Authors:** 


### BRISTOL MEDICO-CHIRURGICAL JOURNAL

*(Incorporating the Medical Journal of the South-West)*

Established 1883

VOL. 80 (iv) October 1965 NO. 298

### Contents

A REVIEW OF LIQUOR AMNII ANALYSES DURING PREGNANCY *R. A. Branch* 61

TRANQUILLIZING AND THYMOLEPTIC DRUGS *R. E. Hemphill and J. E. Barber* 67

IS AN INTENSIVE CARE UNIT REALLY NECESSARY? *P. J. F. Baskett* 82

NEONATAL SEPTICAEMIA . . . *Clinico-Pathological Conference* 87

NOTES AND NEWS . . . . . . . . . . . 95

INDEX TO VOL. 80 . . . . . . . . . . . 97

*PUBLISHED QUARTERLY*

*ANNUAL SUBSCRIPTION* 16/- *POST FREE*

BRISTOL MEDICO-CHURURGICAL JOURNAL
PUBLISHED UNDER THE AUSPICES OF THE

### *Bristol Medico-Chirurgical Society*

The Society was founded in 1874. In 1883 publication of this Journal began, and has continued quarterly since then. During the years 1949 to 1962 it appeared under the title of "The Medical Journal of the South West".

The Society founded a medical library in 1890, which in 1892 was combined with that of the Bristol Medical School. This became the University of Bristol Medical Library in 1925, and an agreement in that year confirmed the privilege of Members to use the University Medical Library.

---

### *Editorial Committee*

Dr. N. J. Brown, Southmead Hospital, Bristol. *Joint Editor.*

Dr. A. B. Raper, Department of Pathology, Bristol Royal Infirmary. *Joint Editor.*

Dr. J. Macrae, Ham Green Hospital.

Dr. R. C. Wofinden, Central Clinic, Bristol.

Mr. A. E. S. Roberts, Medical Library, University of Bristol. *Secretary.*

*Local Correspondents*

*Bath* . . Mr. A. D. Bateman, 3 The Circus, Bath.

*Exeter* . . Dr. L. N. Jackson, Mount Jocelyn, Crediton, Devon.

*Gloucester* . Dr. R. F. Jarrett, 9 College Green, Gloucester.

*Plymouth* . Dr. C. R. Croft, Lexden, Hartley Av., Plymouth.

*Taunton* . Dr. M. McAlley, 1 The Crescent, Taunton.

*Torquay* . Dr. A. Robinson Thomas, 20 Devon Square, Newton Abbot, Devon.

*Truro* . . Dr. F. D. M. Hocking, St. Andrews, Perranporth, Cornwall.

*Yeovil* . . Mr. J. A. Vere Nicoll, Knapp House, Yeovil, Somerset.

### NOTICE TO CONTRIBUTORS

Original articles are invited, provided they have not been published elsewhere. Notes of forthcoming events, meetings held, degrees conferred, staff appointments, and other local news are welcomed. The editorial committee reserves the right to make literary corrections.

Illustrations should always be supplied ready for reproduction. All re-touchings or other operations required will be charged to the author. Line drawings should be drawn in Indian ink. We regret that we must ask authors to pay for making blocks, if this is necessary.

J. W. ARROWSMITH LTD., WINTERSTOKE ROAD
BRISTOL.

### *Notes and News*

*Obituary:* Dr. William Bain.
Col. Thomas Parr.

The following have been elected F.R.C.P.:
J. A. James
H. G. Mather
A. B. Raper
A. E. A. Read

A. J. Buller, B.Sc., M.B., B.S. (Reader in Physiology, King's College, London) has been appointed Professor of Physiology in the University of Bristol.

Neville R. Butler, M.D., F.R.C.P., D.C.H. (Physician, Hospital for Sick Children, Great Ormond Street, London, and Senior Lecturer in Child Health, Institute of Child Health, University of London) has been appointed Professor of Child Health in the University of Bristol.

Examination results, University of Bristol:
*M.D.:* R. E. Midwinter.
*Ph.D. in the Faculty of Medicine* (Veterinary School): J. R. Baker, J. S. E. David, R. H. C. Penny.
*M.B., Ch.B.*

With Second Class Honours:
J. E. Bowerman,
R. A. Branch (*with Distinction in Public Health*)
C. J. F. Davis
D. A. Griffiths
R. J. T. Jarvis

Pass:
C. R. L. Allwood
Dianne M. N. Bateman
M. M. Ben Halim
I. E. Burton
R. J. Chandler
Dominica G. Evans
Y. Frempong
P. R. Geddes
Christine M. Gilman
W. T. Hill
P. A. Johnson
M. J. Kneebone
A. H. Lloyd
A. L. H. Long
Jennifer J. Lovett
D. G. Lytton
J. E. D. Meads
Brenda Moore
C. J. Moran
C. N. Obioha
A. C. Parsons
Angela J. Puzey
B. Rasaiah
Juliet M. Rogers
G. J. Spearing
Louise A. Stephens
J. M. Turner
G. M. Wakley
I. S. Waring
Josephine Wright

**WEST OF ENGLAND**
**CHILD HEALTH GROUP**

1965–1966

CHAIRMAN: DR. S. D. V. WELLER

| *Date and Time* | *Subject* | *Speaker* | *Place* |
|---|---|---|---|
| Thursday 7th October 8.15 p.m. | Some Metabolic Problems in Childhood | Dr. D. Burman, Consultant Paediatrician | Lecture Theatre, Children's Hospital, Bristol. |
| Tuesday 9th November 6 p.m. | Some Aspects of Child Training in Ghana | Dr. Barrington Kaye, Head of Education Department, Redland College, Bristol | ,, |
| Thursday 2nd December 8.15 p.m. | The Present Management of Spina Bifida Cystica | Mr. D. G. Calvert, Senior Registrar, Southmead Hospital | ,, |
| Tuesday 11th January 6 p.m. | Interesting Adolescents | Dr. M. P. S. Seacome, Senior Medical Officer, Maternal and Child Welfare, Gloucestershire | ,, |
| Thursday 3rd February 8.15 p.m. | The Challenge of the Perinatal Period | Professor Neville Butler | ,, |
| Tuesday 8th March 6 p.m. | The Immigrant Child: Some Social Factors | Miss Nadine Peppard, Advisory Officer, National Committee for Commonwealth Immigrants | ,, |
| Saturday 7th May | Clinical Meeting | | The Nuffield Orthopaedic Centre, Oxford |

A. L. SMALLWOOD,
Hon. Secretary,
Department of Public Health,
Tower Hill, Bristol 2.

### INDEX TO VOLUME 80

### SUBJECTS

Appointments, 17, 57
Arhinencephaly, 13
Ataxia in myxoedema, 1
Barbiturate poisoning, 29
Cytology, cervical, 23
Cytomegalic inclusion disease, 53
Habitual abortion—chromosomes, 5
Intensive care unit, 82
Librarian in America, 19
Liquor amnii and Rh disease, 61
Mongolism, 7
Mumps arthritis and prostatitis, 27
Neurosurgery, 42
Placental arteriography, 50
Pridie, Kenneth; an appreciation, 37
Pseudomonas septicaemia, 87
Tranquillizers, 87

### AUTHORS

Ashworth, B., 1
Barber, J. E., 67
Baskett, P. J. F., 82
Branch, R. A., 61
Cornes, J. S., 23
Cree, G. E., 27
Cullen, S. A., 13
Draisey, T. F., 13
Eyre-Brook, A. L., 37
Faint, S. A., 13
Hemphill, R. E., 67
O'Connell, N. D., 50
Phillips, D. G. 42
Roberts, A. E. S., 19
Short, P. W., 7
Way, V., 23
Wingate, L., 5
Wingate, M. B., 23, 50

*Printed by*
J. W. Arrowsmith Ltd., Bristol

